# Comprehensive transcriptome analysis of early male and female *Bactrocera jarvisi *embryos

**DOI:** 10.1186/1471-2156-15-S2-S7

**Published:** 2014-12-01

**Authors:** Jennifer L Morrow, Markus Riegler, A Stuart Gilchrist, Deborah CA Shearman, Marianne Frommer

**Affiliations:** 1 Hawkesbury Institute for the Environment, University of Western Sydney, Locked Bag 1797, Penrith, NSW 2751, Australia; 2 Evolution and Ecology Research Centre, School of Biological, Earth and Environmental Sciences, University of New South Wales, Sydney, NSW 2052, Australia

**Keywords:** tephritid fruit flies, *Bactrocera jarvisi*, RNA-Seq, differential expression

## Abstract

**Background:**

Developing embryos are provided with maternal RNA transcripts and proteins, but transcription from the zygotic nuclei must be activated to control continuing embryonic development. Transcripts are generated at different stages of early development, and those involved in sex determination and cellularisation are some of the earliest to be activated. The male sex in tephritid fruit flies is determined by the presence of a Y chromosome, and it is believed that a transcript from the Y-chromosome sets in motion a cascade that determines male development, as part of the greater maternal to zygotic transition (MTZ). Here we investigate the poly(A^+^) transcriptome in early male and female embryos of the horticultural pest *Bactrocera jarvisi *(Diptera: Tephritidae).

**Results:**

*Bactrocera jarvisi *embryos were collected over two pre-blastoderm time periods, 2-3h and 3-5h after egg laying. Embryos were individually sexed using a Y-chromosome marker, allowing the sex-specific poly(A^+^) transcriptome of single-sex embryo pools to be deep-sequenced and assembled *de novo*. Transcripts for sixteen sex-determination and two cellularisation gene homologues of *Drosophila melanogaster *(Diptera: Drosophilidae) were identified in early embryos of *B. jarvisi*, including transcripts highly upregulated prior to cellularisation. No strong candidates for transcripts derived solely from the Y chromosome were recovered from the poly(A+) fraction.

**Conclusions:**

*Bactrocera jarvisi *provides an excellent model for embryonic studies due to available Y-chromosome markers and the compact time frame for zygotic transcription and the sex-determined state. Our data contribute fundamental information to sex-determination research, and provide candidates for the sourcing of gene promoters for transgenic pest-management strategies of tephritid fruit flies.

## Background

Early stages of embryonic development involve large changes to the RNA transcript profile, as maternal transcripts, deposited during oogenesis, are targeted for degradation, and activation of the zygotic genome takes place. In *Drosophila melanogaster*, egg activation is triggered by osmotic and physical stimulation and occurs independently of fertilisation. Proteins such as SMAUG (SMG) and microRNAs are required to regulate degradation of maternal mRNAs in the developing embryo [1-3]. At least 30% of the transcripts in the *D. melanogaster *early embryo have the distinctive expression profile of maternal transcripts and about two thirds of these decrease markedly over the first 6.5h of development [4].

During these early stages, transcription from the zygotic genome must be initiated. Activation of zygotic transcription is controlled, in part, by the *zelda *(*zld*) protein, which interacts with specific heptamer motifs (TAGteam sites) located in the regulatory regions upstream of genes targeted for early, pre-blastoderm transcription [5,6]. Some of the earliest genes to be transcribed are involved in sex determination, such as *sisterless A *(*sisA*) during nuclear cycle 8 [7]. From cycle 11, cellularisation genes including *serendipity α *(*sryα*), *nullo, bottleneck *(*bnk*) and *slow as molasses *(*slam*) are activated [8-11].

The genes of the sex-determination pathway are highly conserved in Diptera, most notably at the terminal gene, *doublesex (dsx) *[12], and its upstream regulatory genes *transformer (tra) *[13] and *transformer-2 *(*tra-2*) [14]. In *D. melanogaster*, sex-specific splicing of *tra*, which generates the active TRA protein in females and a non-functional protein in males, is regulated by the *Sex-lethal *protein (SXL). Ongoing production of functional SXL occurs in females because an early, transiently-generated *Sxl *protein is produced only in females in response to the primary signal. The mRNA transcribed from the late promoter is spliced in the female-specific mode only when the early SXL is present. The primary signal that leads to female-specific *Sxl *is transmitted by a combination of X-chromosome-linked signal elements (XSE), namely *sisA, scute *(*sc*), *outstretched *(*os*) and *runt *(*run*), whose protein products are more concentrated in XX females than XY males [15]. These gene products interact with maternal products *daughterless *(*da*), *hermaphrodite *(*her*), *extra-macrochaetae *(*emc*) and *groucho *(*gro*) and the zygotically-expressed autosomal gene *deadpan *(*dpn*) [reviewed in [16]]. Many of these genes have other molecular functions in development, and have been co-opted into a sex-determination regulatory role in *Drosophila *as the apex of the pathway diverged from other Diptera.

In contrast to *Drosophila, Sxl *transcripts are not sex-specifically spliced in other Diptera including tephritid species [17,18] and house fly [19], and thus have no clear role in sex-determination. Homologues for some of the genes involved in regulation of *Sxl *in *D. melanogaster *have been identified in EST libraries of the tephritid fruit fly *Ceratitis capitata *[20]. Transcript expression analysis in unfertilised eggs and early developing embryos of *C. capitata *demonstrated the dynamic expression profile of these transcripts during early development [21]. Homologues of the principal sex-determination genes *tra, tra-2 *and *dsx *have been sequenced in *Bactrocera *fruit flies [17,22-24], as has *Sxl*, which differs again from the *Drosophila *model by its maternal deposition in the egg. *Sxl *is also zygotically transcribed in the pre-blastoderm embryo in fruit flies of both genera *Bactrocera *and *Ceratitis *[21,24].

For non-drosophilid insects, in contrast to the well-studied model species *Drosophila*, different modes of regulation of the early stages of development and activation of the sex-determination pathway have been identified. In many dipterans, it appears that zygotic transcription from the Y-chromosome enables the female-specific sex-determination gene transcripts, which are part of the maternal mRNA complement, to be replaced by male-specific transcripts, thus resetting cell memory [25]. This putative Y-chromosome transcript is the *Dominant Male Determiner *(*M*), which has yet to be characterised, but the most likely target appears to be the TRA/TRA-2 based spliceosome, thereby effectively prohibiting the female-specific splicing of both *tra *and *dsx *pre-mRNA [24]. In *Bombyx mori *(Lepidoptera) with a ZZ:ZW sex-determination system where females are the heterogametic sex, a piRNA transcribed from the W chromosome has been identified as the dominant feminising factor [26].

*Bactrocera tryoni *or Queensland fruit fly is the major fruit fly pest species in Australia, exhibiting a wide host fruit range and geographic distribution; it is the primary target in Australia of pest management strategies, including sterile insect technique [SIT; [27]] and potentially incompatible insect technique [IIT; [28]]. *Bactrocera jarvisi *is a tephritid pest endemic to the north and east coast of Australia but has a narrower host range compared to that of *B. tryoni. B. jarvisi *is extremely useful in research because of its tractable laboratory maintenance, ability to form fertile hybrids with *B. tryoni*, and its possession of two Y-chromosome genetic markers, fundamental for discerning male and female embryos [24,29]. These features were exploited to perform qRT-PCR expression analysis of genes, previously identified as *D. melanogaster *sex-determination and cellularisation genes, using single, sexed embryos of both *B. jarvisi *and a *B. tryoni *line carrying the introgressed *B. jarvisi *Y-chromosome [24], from 1h to 9h after egg laying (AEL). The demonstrated reciprocal compatibility of the male determiner in *B. jarvisi *and *B. tryoni *[29] anticipates strategies for optimising SIT and IIT in *B. tryoni*, such as the development of genetic sexing constructs [30] and embryonic lethality [31]. With established germ-line transformation protocols [32], these strategies may be transferable between species, supported by access to whole genome assemblies for both *B. tryoni *(GenBank accession No. JHQJ00000000) and *B. jarvisi *(Gilchrist *et al.*, unpublished).

The embryonic progression of *B. jarvisi *from fertilisation to cellularisation takes seven hours. Expression analysis of *Sxl *and *slam *demonstrated that zygotic transcription has begun by 4h post fertilisation, and affects sex-specific transcripts from 5h in *B. jarvisi *and from about 8 hours in *B. tryoni *laboratory lines [24]. This tight time period of zygotic transcription and sex determination adds to the utility of *B. jarvisi *as a model for early embryonic studies. Therefore, we chose to analyse poly(A^+^) transcriptome data from sexed *B. jarvisi *embryos of 2-3h and 3-5h AEL to investigate three aspects of early development. One focused on the expression of sex-determination genes important to the *Drosophila *sex-determination pathway. The aim was to get a broader insight into the expression profiles of genes that have been co-opted to regulate the sex-determination pathway upstream of the primary genes conserved across tephritids and drosophilids. Secondly, we searched the *B. jarvisi *assembly for genes showing early zygotic expression in *D. melanogaster*, focusing on cellularisation genes, to identify homologues possessing early-acting promoters; and thirdly we used differential expression analysis to search for genes expressed differentially in male and female embryos, including candidate Y-chromosome transcripts, which are not maternally deposited and are only transcribed in males.

## Methods

### Fly Rearing

*B. jarvisi *(BJ) laboratory stock was originally sourced from the Queensland Department of Agriculture, Fisheries and Forestry, Australia, and maintained at University of New South Wales and then University of Western Sydney at constant 25°C, natural light and on artificial larval diet [33]. Flies were fed protein in the form of yeast hydrolysate and sugar one week after emergence, and two to four weeks later gravid females were induced to lay eggs into larval diet covered by perforated parafilm.

### Embryo collection and RNA and DNA extraction

Embryos of two different age ranges were selected for sequencing: 2-3h and 3-5h after egg laying (AEL). For each time period, it was necessary to separate male and female embryos based on a molecular marker on the Y-chromosome, as there are no morphological characters to distinguish the sex of early embryos. Each embryo was individually processed for RNA extraction and DNA was isolated from these embryos for designation of sex. Embryos for RNA and DNA extraction were collected from *B. jarvisi *females by inducing egg laying through perforated parafilm coated with apple juice for a period of 1h (first egg batch discarded), and then placed on moist fabric and maintained at 25°C and 70% humidity for the appropriate time. Individual embryos were placed in microcentrifuge tubes, 100µL TRIsure (Bioline Australia) added and embryos crushed using microtube pestles (SSI). Samples were incubated at room temperature (RT) for several minutes and then stored at -80°C.

Extraction of RNA from single embryos followed the TRIsure method, scaled down to 100µL TRIsure per embryo and further modified for use with Phase lock gel tubes (PLG; 5PRIME, Germany). Here, 40µL DEPC-H_2_O and 70µL chloroform (Sigma, St Louis, MO, USA) were added to the tissue in TRIsure before transferring to the PLG tubes and centrifuging for 5min at 12000g at RT, as per the PLG tube manufacturer's recommendations. The upper aqueous phase was pipetted into fresh tubes containing 5µg glycogen (Roche) and 70µL isopropanol (Sigma) and mixed. The samples were incubated at RT for 10min, then at -20°C for several hours, before centrifugation at 12000g at 4-8°C for 10min. The supernatant was decanted and the RNA was washed twice in 75% ethanol, air dried and resuspended in 15µL nuclease-free water. RNA integrity was examined by non-denaturing agarose gel electrophoresis of 1µL of each 15µL sample. Samples with poor yield or degraded RNA were discarded; acceptable samples were pooled (see below) and stored at -80°C until required.

DNA is found in the interphase of the TRIsure / Chloroform mix after centrifugation, below the gel phase. With the aqueous RNA layer removed, 1M Tris at pH 7.6-8.0 was added to the organic phase, mixed and incubated at RT for more than 15min. Following centrifugation for 10min at 12000g, the aqueous layer containing DNA was transferred to a new tube, ethanol precipitated and resuspended in 10µL nuclease-free water.

Multiplex PCR to designate sex was performed on the DNA from each embryo under conditions reported in Morrow *et al*. [24], using BjY2traB and BjY2traDrev primers to amplify the Y-chromosome fragment (227bp) and primers SxlRTFor1 and SxlRTRev1 to amplify a 280bp product from both sexes (see Additional File 1). When multiplex PCR failed, single target PCR with primers BjY2traA and BjY2traDrev (311bp amplicon) and *Sxl *(as above) was carried out. Many samples failed to amplify with any set of primers probably due to the low copy number; these were discarded.

RNA preparations from the two time points, following sex designation, were pooled to create two male samples at 3-5h AEL (26 and 23 embryos), two female samples at 3-5h AEL (27 and 17 embryos), two male samples at 2-3h AEL (28 and 24 embryos) and two female samples at 2-3h AEL (12 and 24 embryos). Pooled samples were ethanol precipitated, washed six times in 75% ethanol and resuspended in 30µL DEPC-H_2_O. Quality of the RNA preparations was ascertained by Nanodrop spectrophotometry, Qubit DNA and gel electrophoresis (Additional File 2). Poly(A^+^) selection, reverse transcription and library construction using TruSeq RNA LT kit followed the Illumina RNA-Seq protocol and were performed at the Next Generation Sequencing Facility, Hawkesbury Institute for the Environment, UWS (Richmond, Australia). Eight paired-end libraries (300bp), comprising the two female samples and two male samples at both 2-3h AEL and 3-5h AEL time points, were sequenced on two lanes of Illumina HiSeq 1500. The sequencing output of 2 × 100bp was demultiplexed and provided as two paired fastq files for each sample.

### Transcriptome assembly and annotation

The raw sequence data were quality trimmed and filtered using CLC Genomics Workbench ver.6 (CLCbio), with parameters allowing 2 ambiguous nucleotides, minimum read length of 50 nucleotides and error probability limit of 0.05 applied to Phred quality scores according to the modified Mott trimming algorithm (see CLC Genomics Workbench v6 manual). *De novo *assembly of reads from all eight samples combined was performed with CLC Genomics using default parameters (herein referred to as "CLC assembly"). Within the CLC workbench, the entire CLC assembly was queried against the NCBI non-redundant (nr) protein database (downloaded October 2013) using blastx (cut-off E-value 1E-3). The subset of contigs with E-value cut-off of 1E-3 were then used as a query in blastx searches of the NCBI nr protein database followed by gene ontology assignment, implemented in Blast2GO [34].

The CLC assembly was interrogated for twenty-eight genes involved in sex-determination processes in *Drosophila *spp. (GO:0007530) and four genes involved in cellularisation. First, the genes were sought amongst the top BLAST hits and accepted if the E-value was <1E-3. For those genes that did not find a match to *B. jarvisi *sequences in the CLC assembly, *D. melanogaster *and *C. capitata *sequences from GenBank were aligned in Mega 5.05 [35], and, where possible, a highly conserved section of the homologous *C. capitata *gene was used in a motif search of the CLC assembly contigs. The contigs with matches were then realigned with the *D. melanogaster *or *C. capitata *sequences in Mega 5.05 to confirm homology and to determine the length of the ORF. Consensus sequences for genes with multiple contigs were compiled for expression analysis.

### Differential expression

RNA-Seq analysis was performed on the CLC assembly within CLC Genomics Workbench. The trimmed paired reads from each sample were mapped to the CLC assembly using default parameters which include minimum length (0.9) and similarity (0.8) fractions and expression values reported as RPKM (Reads Per Kilobase of exon model per Million mapped reads). Patterns of expression of known *D. melanogaster *sex-determination gene homologues (*Sxl, tra, tra-2 *and *dsx*) from qRT-PCR were compared to the RNA-Seq output. The cellularisation gene *slam *was shown to be transcribed between three to four hours (AEL) by qRT-PCR analysis [24] and the *sisA *transcript was identified in this study (see results). In *D. melanogaster, slam *is expressed in nuclear cycle 11 and *sisA *is transcribed in nuclear cycle 8. The expression levels of these genes were assessed to determine if zygotic transcription was proceeding in the 2-3h embryos, and to determine an appropriate RPKM value threshold, over which expression values are likely to indicate maternally derived transcripts.

Differential expression comparisons were run on fourteen combinations of the eight samples across time and sex, and the RPKM values were quantile normalised and fold changes calculated. The proportions-based (Baggerley's) test was applied to normalised expression values, differences were selected when false discovery rate (FDR) -corrected p < 0.001. To investigate zygotic expression, differential expression output was filtered to select transcripts up-regulated in 3-5h versus 2-3h embryos and male versus female embryos. Selections were filtered further by restricting the mean normalised RPKM values in the baseline sample to a variable number, based on assessment of *slam *and *sisA *transcript expression.

## Results

A developmental time series of *B. jarvisi *transcript levels of homologues of the *D. melanogaster *genes, *Sxl, tra, tra-2, dsx *and *slam*, enabled us to choose two developmental time periods as targets for transcriptome analysis [24]. The period 2-3h AEL was selected because it fell in the phase as zygotic transcription was just beginning, while containing enough nuclei to confidently determine the sex of the embryo; and 3-5h AEL to cover the phase when zygotic transcription was proceeding, including the time when *M *was expected to be active.

### Transcriptome assembly

The Illumina RNA-Seq output comprised eight *B. jarvisi *samples with a range of 53 million to 112 million reads prior to quality trimming and filtering (Additional File 3). The filtered sequence reads from all eight samples were assembled *de novo *by CLC Genomics and produced 61,223 contigs of average size 699bp (Table 1). The majority of contigs were between 200 and 500bp (64.6%), while 34.2% were over 500bp in length. Homology searches using blastx (NCBI nr protein database, October 2013) returned 23,518 sequences (E-value < 1E-3), including 260 (1.1%) sequences matching *Bactrocera *species; 16,246 (69.1%) matching sequences from *Ceratitis *spp., mostly from *C. capitata *(16,209); and 2,596 (11.0%) sequences matching *Drosophila *species. Of those sequences homologous to *Bactrocera*, the majority corresponded to *B. dorsalis *(156), *B. oleae *(64) and *B. tryoni *(29).

**Table 1:** CLC *de novo *assembly of transcripts from all eight libraries combined.

Summary Statistics	Count	Average length	Total bases	Nucleotide distribution	Count	Frequency
Reads	604,738,912	99.24	60,014,654,938	Adenine (A)	13,031,025	30.40%
Matched	476,830,889	99.24	47,320,571,906	Cytosine (C)	7,923,401	18.50%
Not matched	127,908,023	99.24	12,694,083,032	Guanine (G)	7,925,335	18.50%
Reads in pairs	418,126,768	154.99		Thymine (T)	13,018,385	30.40%
Broken paired reads	58,704,121	98.54		Any nucleotide (N)	899,616	2.10%

**Contig measurements**	**Length**					

Count	61,223					
N75	434					
N50	1,138					
N25	2,697					
Minimum contig (bp)	114					
Maximum contig (bp)	17,400					
Average (bp)	699					
Total (bp)	42,797,762					

The CLC assembly dataset was too large to annotate using Blast2GO, therefore the subset of 23,518 contigs with E-values lower than 1E-3, was uploaded into Blast2GO [34] for BLAST matching and gene ontology (GO). BLAST using the September 2013 NCBI invertebrate reference library matched 19,449 sequences (82.7%); the top hits distributed primarily among *Drosophila *spp., most prominently *D. virilis, D. mojavensis *and *D. melanogaster *(Additional File 4).

**Figure 1:**
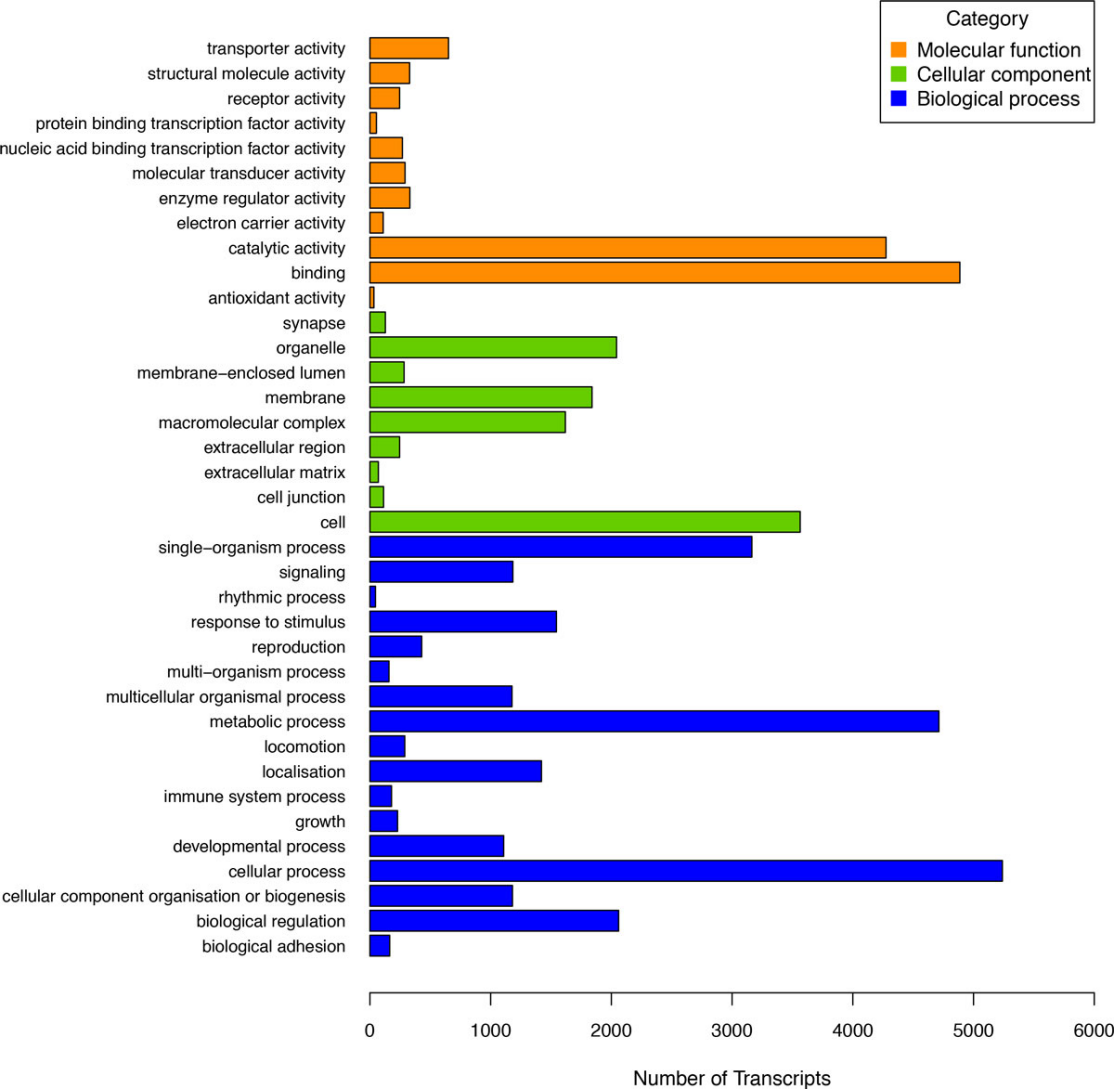
**Classification of the gene ontology (GO) terms for the *B. jarvisi *transcriptome**. GO was implemented in BLAST2GO on a subset of the CLC assembly comprising 23,518 contigs. Of these, 8,864 transcripts (45.6%) were classified into 37 functional groups.

Gene ontology (GO) analysis annotated 8,864 transcripts (45.6%), categorised into 37 functional groups within the classes molecular function, cellular component and biological process (Figure 1). The largest representations were in "binding" and "catalytic activity" (molecular function), "cell" (cellular component) and "cellular process", "metabolic process" and "single-organism process" (biological process). Within biological processes, 1,108 sequences mapped to developmental processes (GO:0032502); 274 sequences were developmental processes involved in reproduction (GO:0003006) and 13 sequences mapped to sex determination (GO:0007530).

### RNA expression analysis and validation

**Figure 2:**
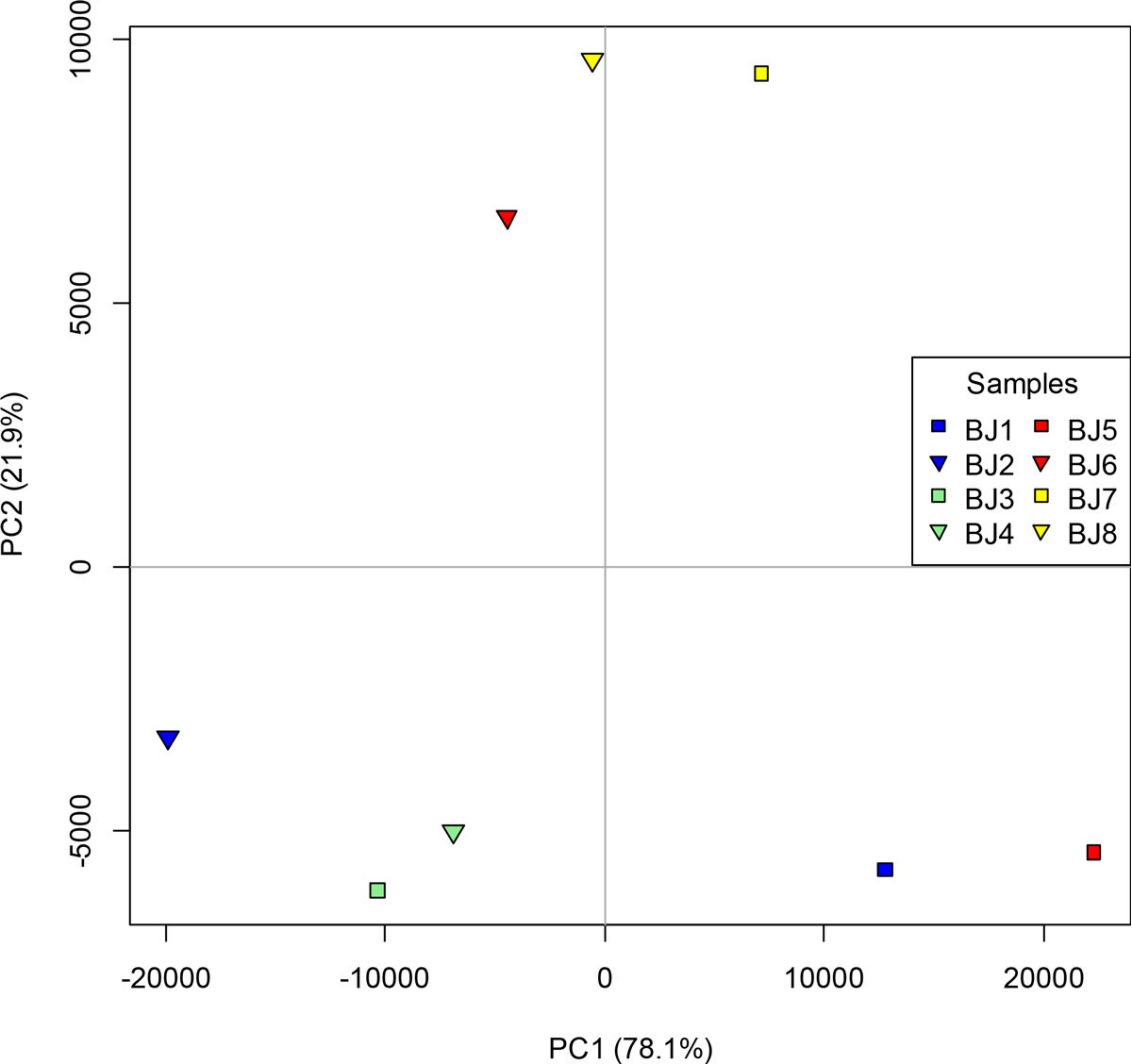
**Principal co-ordinates analysis of the eight transcriptome samples mapped to the CLC assembly**. Samples are male 3-5h (blue), female 3-5h (green), male 2-3h (red) and female 2-3h (yellow).

Within the RNA-Seq module of CLC Genomics, each trimmed library was mapped to the CLC assembly and levels of expression were recorded as normalised RPKM values. Validation of the assembly and mapping was performed. Principal components analysis of the eight samples showed samples clustering according to age, except for two samples, BJ1[male 3-5h] and BJ5[male 2-3h], which clustered together and not with their replicates (Figure 2). To investigate this discrepancy, the comparative expression levels over the two time points for the genes *Sxl, tra, tra-2, dsx *and *slam *[24] served as controls to validate the RPKM values. As there is no difference between male and female embryos in expression pattern for *Sxl, tra-2 *and female-specific *tra *and *dsx *over the first 5h, differential expression across the two developmental periods, irrespective of sex, was examined. However, the results for *Sxl *(maternal and zygotic expression) and *slam *(zygotic only) were most useful because both exhibit an increase in transcript abundance over this time course [24]. *Sxl *RPKM values indicated that BJ1[male 3-5h] had low expression levels equivalent to the 2-3h samples, rather than the higher levels of the other 3-5h samples. The RPKM values for *slam*, a zygotically transcribed gene validated by RT-PCR, were low in BJ1[male 3-5h] (36 RPKM) compared to the other three 3-5h samples (299-461 RPKM), and were very low in three 2-3h samples (0.25-2.02 RPKM) with the fourth 2-3h sample, BJ8[female 2-3h] showing a somewhat higher level (13 RPKM). The difference in RPKM values over time periods was not significant for *slam*, unless BJ1[male 3-5h] alone or BJ1[male 3-5h] and BJ5[male 2-3h] were excluded (FDR < 0.001). A similar phenomenon was also observed for *sisA, run, gro, female lethal d *(*fl(2)d*), *emc *and *dpn *(see sex-determination gene analysis, below), where each gene exhibited increased transcription in the other three 3-5h embryos (and significant up-regulation following exclusion of BJ1[male 3-5h] and BJ5[male 2-3h]; FDR<0.001).

Taken together, these results suggest two features of the samples that may cause discrepancies (Figure 2): firstly, the age range of one hour in the 2-3h samples and 2 hours in 3-5h samples may have introduced variability, especially in the expression of early or rapidly increasing transcripts, such as *slam*, but may also have resulted in detection of early zygotic activity of *emc, dpn *and *run *(for example, if sample BJ8 included some more developed embryos). Secondly, the expression levels of BJ1[male 3-5h] transcripts appeared to result from either a preponderance of embryos at the earlier end of the range, or from a sample in which many embryos did not survive to develop to the full 3-5h, thus producing a weaker increase in expression levels for those genes undergoing zygotic transcription, or a higher apparent expression value for maternal transcripts undergoing degradation in older embryos. Therefore, differential analysis was performed both with and without the BJ1[male 3-5h] and BJ5[male 2-3h] samples, employing 14 combinations to identify changes in transcript expression. The number of contigs differentially expressed (FDR<0.001), shows clearly that when time comparisons were undertaken, greater differences were recovered in the absence of samples BJ1[male 3-5h] and BJ5[male 2-3h], with BJ1 responsible for the greater impact. As a result, and to maintain consistency, the analyses of "*Data mining for differentially expressed transcripts*", below, were performed with and without BJ1 and BJ5.

### Sex-determination gene expression

The CLC assembly was examined for the 28 *Drosophila *genes categorised as sex determination group (GO:0007530). Using the blastx annotation of the CLC assembly, the 13 contigs classified as sex-determination in the BLAST2GO pipeline and additional motif searches, the contigs were screened and 20 genes secured a contig match (Additional File 5A, Table 2A). The matching contigs were verified by alignment with the database nucleotide sequence and the ORF determined. This process also helped to assess the quality of assembly through identification of complete or partial open reading frames (ORFs). Only ten transcripts contained a complete ORF in a single contig and two more single contigs covered over 90% of the ORF (for *Sxl *and *fl(2)d*). Eight genes were traversed by two to 11 contigs which covered more than 90% of the ORF (Additional File 5A), thus consensus sequences were produced by combining these contigs manually. The limitations of assembly in some cases appeared to be the result of different variants or alleles, but may be due to insufficiently stringent quality control.

Four of these genes - *Sxl, tra-2*, female-specific *tra *and *dsx *- are recognised maternal transcripts in *B. jarvisi *that exhibited either no substantial change over the 2-5h period or, for *Sxl*, increased expression between 3 and 4h [24]. RNA-seq of 2-3h and 3-5h embryos detected high levels of these transcripts, except for *dsx *where low levels (normalised RPKM<1) of not always complete transcripts were detected in all samples. No significant change in expression from one time sample to the next was recorded. Other *Drosophila *sex-determination genes detected by BLAST homology and expressed in 2-3h embryos are likely to represent maternally derived transcripts. This includes *da, fl(2)d, fruitless*F (*fru*), *gro, hopscotch *(*hop*), *Mes-4 *(*Mes-4*), *ovarian tumour *(*otu*), *sans fille *(*snf*) and *virilizer *(*vir*) (Table 2A). Other transcripts that are first transcribed in the zygote include *dpn *and *emc*, which have low RPKM in 2-3h samples (0.25-1.06 and 0.44-2.5 respectively); for *emc*, the apparent early zygotic transcription contrasted with its maternal deposition in *Drosophila*.

**Table 2:** Expression profiles of the 20 sex-determination (**A**) and three cellularisation (**B**) transcripts identified in the *B*. jarvisi CLC assembly. Expression in each sample BJ1 - BJ8 is reported as the normalised RPKM value from the comparison of early samples (BJ5-8) to late samples (BJ1-4). Also refer to Additional File 5.

				"early" samples				"late" samples				Significant up or down-regulation Comparison		Validated expression profile#		
				**male**		**female**		**male**		**female**						

**A. Sex determination genes**	contig no.	contig size	ORF size	BJ5	BJ6	BJ7	BJ8	BJ1	BJ2	BJ3	BJ4	early vs late	early vs late (exclude BJ1 and BJ5)	Bj	Cc	Dm

*doublesex F*	consensus	1839	321aa	0.42	0.19	0.17	0.22	0.55	0.04	0.09	0.03	ns	ns	M	M	Z
*daughterless*	16154	4053	710aa	3.03	3.39	9.88	9.94	1.51	4.52	4.26	4.53	ns	ns			M
*deadpan*	20953	3365	574aa	0.28	0.25	0.26	1.06	0.95	10.92	11.18	24.97	ns	0.02		Z	Z
*degringolade*	2208	1737	385aa	111.44	116.21	107.56	104.73	149.23	100.76	93.48	100.61	ns	ns			M
*extra macrochaetae*	10611	1230	265aa	0.97	0.44	0.56	2.5	6.82	121.25	123.56	211.04	ns	<0.001			M
*female lethal d*	2854	2694	466aa *	18.97	13.9	12.22	11.07	17.85	40.43	43.89	37.66	0.03	<0.001		M	M
*fruitless*	29049	3175	675aa	0.78	0.57	0.74	0.43	0.62	0.19	0.38	0.08	ns	ns			
*groucho*	3466	4604	726aa	16.34	7.42	13.32	12.69	13.69	51.44	59.52	94.36	ns	<0.001		M	M
*hopscotch*	753	4443	1174aa	53.5	20.8	23.27	19.15	40.31	15	20.17	23.18	ns	ns			
*Mes-4 *	5091	5397	1557aa	57.2	17.96	22.76	16.62	34.22	11.54	18.9	17.46	ns	ns			
*ovarian tumor*	consensus	5276	1221aa *	31.52	22.68	62.4	46.29	22.6	20.46	26.52	16.5	ns	ns			
*ovo (variant1)*	21175	255	86aa *	3.93	2.97	8.77	14.62	1.44	0.09	0.43	0.23	ns	ns			
*ovo (variant 6)*	consensus	3076	739aa	12.66	11.2	58.05	51.86	5.85	0.83	0.61	0.86	ns	ns			
*runt*	consensus	2399	485aa	0.74	0.22	0.13	5.4	11.8	65.22	58.82	36.12	0.04	<0.001			Z
*sans fille*	7153	1421	150aa	35.59	80.26	75.06	85.98	43.71	65.1	55.76	50.27	ns	0.008		M	
*scute*	consensus	817	272aa *	0.11	0.15	0.12	0.17	0.07	0.05	0.05	0.08	ns	ns			Z
*Sex-lethal*	2265	1667	444aa *	23.93	21.11	23.7	22.69	22.68	38.45	42.67	64.18	ns	0.04	M	M	Z
*sisterless A*	2508	264	89aa *	71.62	39.09	29.59	38.58	89.42	180.25	216.18	294.05	0.03	<0.001		Z	Z
*transformer F*	consensus	3793	422aa	27.3	22.76	18.43	18.49	28.18	17.12	17.29	21.22	ns	ns	M	M	M
*transformer-2*	138	3165	251aa	48.16	44.99	41.74	44.01	47.47	37.15	37.52	39.39	ns	ns	M	M	M
*virilizer*	consensus	5999	1878aa	27.4	31.59	38.11	36.09	30.89	28.63	32.69	27.83	ns	ns			Z

**B. Cellularisation genes**																

*nullo*	consensus	1332	211aa	1.15	2.52	0.13	15.34	8.59	162.77	125.03	45.74	ns	0.04			Z
*serendipity α*	9105	5007	615aa *	13.31	11.32	10.66	9.64	12.42	4.22	3.5	4.31	ns	ns		Z	Z
*slow as molasses*	3824	5366	1568aa	2.02	0.76	0.25	13.35	35.87	460.96	425.02	299.29	ns	<0.001	Z	Z	Z

*Partial ORF

#Experimentally validated in *B. jarvisi *(Bj), *C. capitata *(Cc) and *D. melanogaster *(Dm); maternal (M) or zygotic (Z) expression

### Cellularisation genes

Some genes involved in cellularisation in *Drosophila *species are activated early in embryonic development. No *slam *was detected in 0-3h embryos by qRT-PCR [24], but transcript levels rapidly increased over the next three hours of development. Differential expression of *slam *transcripts revealed a mean RPKM of 4.1 in 2-3h embryos and 305.3 in 3-5h embryos (Table 2B), signifying a substantial increase (76-fold) over the two time periods, but also showing that *slam *is zygotically expressed at low levels earlier than 3h. Three additional cellularisation genes *sryα, nullo *and *bnk *were sought through BLAST annotation and motif searches of the CLC assembly (Additional File 5B). A potential match was found for *nullo *with 24.6% identity across 213 amino acids with *Drosophila simulans nullo *(Acc. No. DSY44733) and 57% identity over a conserved stretch of 28 amino acids. Other than *nullo *homologues from ten *Drosophila *species, there were no records of *nullo *in the NCBI database. This sequence displayed a similar expression profile as *slam*, with an 18-fold increase over time (Table 2B). Neither *sryα *nor *bnk *were detected through the BLAST annotation, but a motif search of a 50bp fragment of the *C. capitata sryα *gene (Acc. No. FJ460703) identified a single contig with a partial ORF of 615 amino acids, exhibiting 61.6% and 33.5% identity with *C. capitata *and *D. melanogaster sryα *respectively. The expression profile did not emulate that of a zygotic transcript, as transcript levels diminished 2-fold as the embryos aged.

### Data mining for differentially expressed transcripts

For male/female comparisons, differentially expressed transcripts that adhered to criteria of low or zero female expression with higher expression in males (Table 3 comparisons A-G) were only found in comparisons B and D, identifying five and four transcripts, respectively (Table 4). Annotation revealed contaminant fungal, bacterial and ribosomal RNA sequences in seven of them. One 220bp sequence fragment was expressed 23-fold higher in male embryos at 2-3h of age: this sequence matched the *B. dorsalis even-skipped *(*eve*) pair-rule gene. However there was little support for this transcript to be considered a good Y-chromosome candidate, as examination of the raw reads did not reveal either a pseudogene copy or alternative structure of *eve*; and inclusion of male and female data from 3-5h, showed higher expression in females as time proceeds, but a reduction in the male samples over time. To broaden the search for transcripts up-regulated in males, further comparisons were made to distinguish these transcripts; the most useful comparisons were I, L and N (Table 3). The differential expression between the 2-3h embryos and the 3-5h embryos of both sexes (I) revealed significant change of expression in 2,313 transcripts, of which 1,213 were up-regulated in older embryos (FDR-corrected p < 0.001) indicating zygotic transcription. The comparison of BJ2[male 3-5h] and BJ6[male 2-3h] (L) was not replicated, but was used to find the overlap with the sequences from I (I ∩ L). A subset of sequences expressed at no more than 50 RPKM in the 2-3h samples was used to eliminate transcripts that are 20% more abundant than *sisA*, a conservative estimate to minimise the elimination of candidate transcripts that were highly expressed in the 2-3h embryos. As a result, 341 transcripts common to both sets (1,051 and 673 sequences respectively) were extracted. To reduce the inclusion of false positives, comparison N, highlighting up-regulated sequences in older female embryos, was added to exclude those sequences also transcribed strongly in female embryos. The resulting 105 sequences had top BLAST hits to 53 *C. capitata *sequences, 9 *Drosophila*, four transposon-related sequences from other arthropods, and 39 undetermined sequences (E-value > 1E-3 or no match retrieved; Additional File 6 Additional File 7).

**Table 3:** Summary of differential expression comparisons performed in CLC Genomics Workbench. Expression values were normalised and significant difference ascertained by Baggerley's test, with FDR-corrected p value cutoff at 0.1%. Contigs were selected for up-regulation in males over females, and 3-5h embryos over 2-3h embryos using normalised fold-change.

	Comparison	Samples¹	No. of differentially expressed contigs (FDR p < 0.001)	No. up-regulated in males and/or older embryos	Additional filters	No. of transcripts of interest
	**Male/Female comparisons**					
A	Late only (3-5h)	1,2 vs 3,4	2	0		0
B	Late only (3-5h), excl. BJ1	2 vs 3,4	21	9	†mean normalised RPKM of female samples <5	5
C	Early only (2-3h)	5,6 vs 7,8	73	4	†mean normalised RPKM of female samples <5	0
D	Early only (2-3h), excl. BJ5	6 vs 7,8	142	46	†mean normalised RPKM of female samples <5	4
E	Both (3-5h and 2-3h)	1,2,5,6 vs 3,4,7,8	0	0		
F	Both (3-5h and 2-3h) excl. BJ1 and BJ5	2,6 vs 3,4,7,8	0	0		
G	Both (3-5h and 2-3h) excl. BJ1	2,5,6 vs 3,4,7,8	0	0		
	**Time comparisons**					
H	Both (Male and female)	1,2,3,4 vs 5,6,7,8	283	86		
I	Both (Male and female) excl. BJ1 and BJ5	2,3,4 vs 6,7,8	2313	1213	#mean normalised RPKM of 2-3h samples <50	1051*
J	Both (Male and female) excl. BJ1	2,3,4 vs 5,6,7,8	1846	1128	#mean normalised RPKM of 2-3h samples <50	1019
K	Male only	1,2 vs 5,6	14	2		
L	Male only excl. BJ1 and BJ5	2 vs 6	1321	827	#mean normalised RPKM of 2-3h samples <50	673*
M	Male only excl. BJ2 and BJ6	1 vs 5	183	66		
N	Female only	3,4 vs 7,8	1980	862*		
	**Intersections**					
	I ∩ L					341
	(I ∩ L) - (I ∩ L ∩ N)					105

¹ Samples BJ1- BJ8 are abbreviated to 1-8

†Allowed extra expression (RPKM<5) in female samples to cover any accidental inclusion of male embryos

# Determined cutoff to be 20% higher than the mean expression, in 2-3h embryos, of *sisA*, a transcript expressed early in *D. melanogaster*

*Subsets of I, L and N used in intersections

**Table 4:** Differential expression results showing significantly up-regulated transcripts in males over females in 3-5h samples (Comparison B) and 2-3h samples (Comparison D).

Comparison B			Normalized expression values (RPKM)					Top BLAST hit
**Late only (3-5h), excl. BJ1**			**male**	**female**				

**Feature ID**	**Gene length**	**Fold Change (normalized values)**	**BJ2**	**BJ3**	**BJ4**	**NCBI Accession No**.	**E-value**	**Gene / Source**

contig 22537	1546	-109.63	185.79	0.1	3.29	EQB46020	7.85E-55	hypothetical protein CGLO_15009 [Colletotrichum gloeosporioides Cg-14] (fungal pathogen of plants)
contig 26828	407	-90.35	145.56	0	3.22	ELT94824	2.06E-40	hypothetical protein CAPTEDRAFT_122939, partial [Capitella teleta] (Annelid worm)
contig 30721	521	-44.23	68.22	0.39	2.7	EEH16716	2.00E-28	senescence-associated protein [Paracoccidioides brasiliensis Pb03] >gi|225678434|gb|EEH16718.1| (fungus)
contig 47470	424	-100.05	111.08	0.12	2.1	XP_662849	3.93E-26	hypothetical protein AN5245.2 [Aspergillus nidulans FGSC A4] >gi|40742997|gb|EAA62187.1| (fungus)
contig 49000	222	-39.38	131.19	0.96	5.71	EHK21457	3.99E-41	hypothetical protein TRIVIDRAFT_53758, partial [Trichoderma virens Gv29-8] (fungus)

**Comparison D**								
**Early only (2-3h), excl. BJ5**								

			**BJ6**	**BJ7**	**BJ8**			

contig 31948	667	-387.12	64.8	0.1	0.23	CAJ30045	7.17E-28	conserved hypothetical protein [Magnetospirillum gryphiswaldense MSR-1] (bacterium)
contig 3465	234	-20.53	29.21	0.57	2.28	XP_004522331	3.36E-45	PREDICTED: 60S acidic ribosomal protein P0-like [Ceratitis capitata] >gi|20139848|sp|Q9U3U0.1|
contig 41160	210	-70.11	37.85	0.33	0.75	WP_006574328	1.06E-16	hypothetical protein [Pseudoflavonifractor capillosus] >gi|150270408|gb|EDM97731.1(bacterium)
contig 567	220	-23.73	33.44	2.82	0	ACN91520	2.50E-16	eve [Bactrocera dorsalis]

Two *Drosophila *genes involved in the maternal to zygotic transition, *zelda *(also known as *vielfaltig, vfl*) and *smaug*, are both expressed maternally, with *smaug *undergoing rapid degradation during nuclear cycles 3-6 [36]. Homologues of *zelda*, as identified by blastx identity (contig 13,492; Additional file 7), displayed a significant 5.8-fold increase in expression over time. The *smaug *transcript was identified by homology to *C. capitata *(Accession No. XP_004526919), and displayed an 11-fold decrease in transcript levels over the two time periods, emulating the expression profile of *smaug *in *D. melanogaster*.

## Discussion

By utilising next-generation sequencing technology, we sequenced the transcriptome of *B. jarvisi *embryos during two developmental periods prior to blastoderm formation. Notably, by exploiting a molecular marker on the Y-chromosome of *B. jarvisi *males, this early and dynamic developmental period was examined independently in male and female embryos to distinguish transcripts differentially regulated between the sexes and over time. The identification, nucleotide sequence and broad expression profile of genes expressed in the early stages of sex determination provided the primary focus of this study and was approached in two ways: through homology-based identification of validated sex-determination genes from *Drosophila *and homologues from other tephritids including *C. capitata*; and expression-based studies highlighting differential regulation of the poly(A^+^) transcriptome over time and between the sexes. Attention was also given to identification and expression profiles of transcripts involved in the maternal to zygotic transition and cellularisation. This collection of transcriptome data is a substantial addition to the knowledge base of this fruit fly species and other closely-related *Bactrocera *species and it will form a basis for future laboratory-based analyses of gene expression in the early embryo.

### Different roles for sex-determination genes

Sex-specific differences in expression levels of the four XSEs found in *Drosophila *were not observed for these genes in *B. jarvisi*. The four genes, *sisA, sc, os *and *run *are transcribed from both X chromosomes in *Drosophila *females, to communicate the female signal to *Sxl*. Bearing in mind the non-sex-specificity of *Sxl *in tephritids, we identified *B. jarvisi *homologues of *sisA, sc *and *run*, but not *os*, expressed in the early developmental stages; *sisA *and *run *increased 6- and 28-fold respectively over the two periods but did not differ in male and female embryos, and *sc *did not change over time. The X and Y chromosomes of *B. tryoni *are highly heterochromatic and the genes located on the *Drosophila *X chromosome are autosomal in tephritids [37], with no evidence that these genes play a role in sex determination. Of the four *Drosophila *XSEs, *os *is expressed latest (cycle 13) which may account for its absence in *B. jarvisi *embryos, or more likely, the *B. jarvisi *homologue was not sufficiently similar to the known *Drosophila os *sequences. However, while *sisA *is expressed during cycle 8 and *sc *during cycle 9 of *Drosophila *embryos [7], in *B. jarvisi, sisA *was highly expressed even in the younger embryos and may be displaying a rapid increase as development proceeds, and *sc *was expressed very low in both time periods. Transcripts of *sisA *were not detected in *C. capitata *unfertilised eggs [21]. If *B. jarvisi *also conforms to this expression profile, then zygotic transcription of *sisA *is very early, well underway between 2 and 3h AEL. An autosomal sex-determination gene in *Drosophila *expressed in the zygote is *dpn*, which complied with this pattern in *B. jarvisi*, and exhibited more than 30-fold increase in transcript levels from 2-3h to 3-5h AEL.

Maternal transcripts in *Drosophila *include *her, da, gro *and *emc*. Homologous sequence for *her *was not detected in *B. jarvisi *embryos, but *da *and *gro *were detected in 2-3h embryos and *gro *exhibited a significant 6-fold increase over time. In *C. capitata, gro *transcripts were found to be maternal in origin [21]. Although confirmation is needed for the maternal input of *da *and *gro *in *B. jarvisi*, their presence in the 2-3hr transcriptomes would suggest that they are maternal transcripts. The expression profile of *emc *differed markedly from that expected for a maternal transcript and demonstrated in *D. melanogaster*; it displayed a significant 130-fold increase in transcript levels in 3-5h embryos, warranting further investigation of the potential functional role/s of this transcript in the developing embryo of *Bactrocera *relative to *Drosophila*.

### Early zygotic transcripts as a source of promoters and enhancers for transgenic pest control strategies

A partial sequence for a putative homologue of the *D. melanogaster *gene *nullo *was identified by BLAST annotation, coupled with an expression profile emulating a known cellularisation gene, *slam*, which is also expressed from nuclear cycle 11 in *D. melanogaster *[8,11]. The *sryα *transcript, however, undetected in *C. capitata *prior to cellularisation [38] but reported as a maternal contribution by Gabrieli et al. [21], was found in *B. jarvisi *2-3h embryos but had diminished levels in 3-5h embryos. Both *slam *and *nullo*, in addition to *sisA*, which also appeared to be expressed early in *B. jarvisi*, provide candidate promoter regions for use in transgenic constructs in the *Bactrocera*. The promoter/enhancer regions of the *sryα *and *slam *genes have been used to create embryonic lethality systems in *C. capitata *[38] and the *Anastrepha suspensa sryα *homologue has been successfully applied to the construction of embryonic lethal [31] and female-specific lethal transgenes [30].

### Up-regulated transcripts in male embryos

We were unable to confidently discern poly(A^+^) RNA that was more abundant in males and very low in females, and therefore potentially transcribed from the Y chromosome. The Y chromosome is highly heterochromatic [37], therefore few genes may be transcribed and these transcripts may not be collected in the poly(A^+^) fraction of the transcriptome. However, the sequencing of the poly(A^+^) fraction of mRNA was an essential step for *de novo *assembly of male and female transcriptomes in the early *Bactrocera *embryo, providing numerous novel coding sequences with homology to known *C. capitata *and *Drosophila *spp. genes. These reads will add valuable sequence for analysis of the *B. jarvisi *draft genome assembly (Gilchrist *et al*. unpublished). Y-specific transcripts may yet be found by obtaining sufficient coverage to identify the Y-chromosome sequences by subtraction of the genome of female *B. jarvisi *from the male genome. Such an endeavour is underway, thus transcripts from the male samples could be mapped onto the Y chromosome and expression analysis repeated to determine up-regulated Y-specific transcripts as the embryos age.

## Conclusion

To extend the results presented here, the next step will be to utilise whole genome sequence for *B. jarvisi*. Genome-guided transcriptome assembly was not feasible in this time frame, however applying this method may uncover transcripts that are differentially expressed or alternatively spliced or located on the Y-chromosome. The differential analysis of the poly(A^+^) fraction has demonstrated the value of *B. jarvisi *as a study system for early embryonic development and sex determination. However, it has provided no evidence that *M*, the *Dominant Male Determiner*, might be a protein coding gene on the Y chromosome, and leaves open the possibility that any Y-chromosome transcripts initiating male development may be non-protein coding. To identify potential candidates for *M*, it will be necessary to utilise the *B. jarvisi *embryo-sexing protocol and tight developmental time frame to also sequence the non-coding fractions of the transcriptome, that includes many regulatory RNAs [39]. The related pest fruit fly, *B. tryoni*, will soon have an annotated genome available, and so homology to the genes discovered here will allow valuable comparisons between the two species, which are reproductively compatible, such that the Y chromosome can be transferred between species in both directions. This work will be immediately useful for the identification and laboratory testing of cellularisation gene promoters, such as *slam *and *nullo*, for transgenic-based improvements to pest management strategies such as sterile insect technique (SIT) for both *B. jarvisi *and *B. tryoni*.

## Availability of supporting data

Raw sequence reads have been submitted to the NCBI SRA (sequence read archive) database under accession number SRP046518.

## Competing interests

The authors declare that they have no competing interests.

## Authors' contributions

The project was conceived and designed by D.C.A.S., M.F., M.R. and J.L.M.; J.L.M. performed the research, J.L.M. and A.S.G analysed the data; M.R. supplied all materials for sample preparation; J.L.M., M.R., D.C.A.S and M.F. contributed to writing the paper.

## Supplementary Material

Additional File 1 **PCR conditions and primer sequences used in this research**. Click here for file

Additional File 2 **Quality and concentration of total RNA samples for transcriptome sequencing**. Click here for file

Additional File 3 **Results of trimming and filtering poor quality reads in CLC Genomics Workbench**. Click here for file

Additional File 4 **Species distribution of BLAST2GO top matches against a subset of the CLC assembly. A subset of 23,518 contigs were selected by e-value > 1E-3 match to NCBI nr database.** Over 77% of the sequences matched *Drosophila *species. Click here for file

Additional File 5 **Sex-determination and cellularisation genes. A) **The 28 *Drosophila *sex determination genes and the top BLAST hits against the nr database. Only 20 of these genes found a match in the CLC assembly. **(B) **Four cellularisation genes were sought, only *nullo *was found by the top blast hits. Other transcripts were identified by motif searches using *D. melanogaster *or *C. capitata *sequence as the query. Click here for file

Additional File 6 **A list of the 66 contigs up-regulated in older male embryos, after exclusion of contigs also up-regulated in older females**. Normalised RPKM values are from comparison I (Table 3) with top BLAST hits to arthropod sequences from the NCBI nr database. Click here for file

Additional File 7 **A list of the *Bactrocera jarvisi *contigs up-regulated in older embryos with BLAST matches to *C. capitata *and *Drosophila *genes (62 contigs).** These were cross-referenced to FlyBase. The molecular and biological function of these genes and the expression profile in *D. melanogaster *are listed. Many genes have transcription or translation regulatory functions, but are transcribed in both males and females (see Additional File 6). Click here for file
