# Supplementary material for: Comprehensive transcriptome analysis of early male and female Bactrocera jarvisi embryos
Source: BMC Genet. 2014 Dec 1;15(Suppl 2):S7. doi: 10.1186/1471-2156-15-S2-S7 (PMC4255828; doi:10.1186/1471-2156-15-S2-S7)
Supplement: Additional File 1 — PCR conditions and primer sequences used in this research. [file 1471-2156-15-S2-S7-S1.pdf]

Additional File 1. PCR conditions and primer sequences used in this research

PCR protocol	Y-Chromosome marker	Multiplex Y-Chromosome
Reaction volume	10µL	10µL
GoTaq Reaction Buffer	1X	1X
$MgCl_2$	2.5mM	2.5mM
dNTP	200µM each	200µM each
Primer 1	0.375µM	0.375µM
Primer 2	0.375µM	0.375µM
Primer 3		0.375µM
Primer 4		0.375µM
GoTaq DNA polymerase	0.5u	0.5u
DNA Template	2µL	2µL
**Thermal Cycling**	94°C for 5min	94°C for 5min
	94°C for 30s, 60°C for 30s, 72°C for 1min (x35 cycles)	94°C for 30s, 60°C for 30s, 72°C for 1min (x35 cycles)
	72°C for 7min	72°C for 7min
**Primers**	**Sequence 5'-3'**	**Combinations (product size)**
BjY2traA	GAATAGTTATAATG/AGCCCTCGTTCACG	BjY2traA-BjY2traDrev (311bp)
BjY2traB	AATGGGCGTCG/ACGATATTCAAAAG	BjY2traB-BjY2traDrev (227bp)
BjY2traDrev	CACTGGCTTTTC/CCTATTACCACGAC	
SxlRTfor1	AGACAAATTGACGGGCAAACCACG	SxlRTfor1 - SxlRTrev1 (280bp)
SxlRTrev1	GACATGTATTGCTGCGCTTTCGCT	

PCR reagents are from Promega (GoTaq DNA polymerase), Bioline (dNTPs), Macrogen (oligonucleotides); thermocycler is the BioRad DNA Engine Dyad.
