# Supplementary material for: Comprehensive transcriptome analysis of early male and female Bactrocera jarvisi embryos
Source: BMC Genet. 2014 Dec 1;15(Suppl 2):S7. doi: 10.1186/1471-2156-15-S2-S7 (PMC4255828; doi:10.1186/1471-2156-15-S2-S7)
Supplement: Additional File 2 — Quality and concentration of total RNA samples for transcriptome sequencing. [file 1471-2156-15-S2-S7-S2.pdf]

Additional File 2. Quality and concentration of total RNA samples for transcriptome sequencing.

			No. of	Nanodrop			Qubit
Sample ID	sex	age (AEL)	embryos	ng/uL	260/280nm	260/230nm	ng/uL
BJ1	male	3-5h	26	103.8	1.99	2.02	63
BJ2	male	3-5h	23	177.5	2.07	1.74	115
BJ3	female	3-5h	27	193.3	2.09	1.65	119
BJ4	female	3-5h	17	137.5	2.05	1.82	93
BJ5	male	2-3h	28	120.6	2.07	2.04	84
BJ6	male	2-3h	24	208.1	2	1.77	125
BJ7	female	2-3h	24	268	2.09	1.76	104
BJ8	female	2-3h	12	101.3	2.03	1.8	59
