# Supplementary material for: Comprehensive transcriptome analysis of early male and female Bactrocera jarvisi embryos
Source: BMC Genet. 2014 Dec 1;15(Suppl 2):S7. doi: 10.1186/1471-2156-15-S2-S7 (PMC4255828; doi:10.1186/1471-2156-15-S2-S7)
Supplement: Additional File 3 — Results of trimming and filtering poor quality reads in CLC Genomics Workbench. [file 1471-2156-15-S2-S7-S3.pdf]

Additional File 3. Results of trimming and filtering poor quality reads in CLC Genomics Workbench.

Sample ID	Paired reads (fastq files)	Number of reads	Average length	Number of reads after trim	Percentage trimmed	Average length after trim
BJ1	JM1_CAGATC_L007_R1_001/JM1_CAGATC_L007_R2_001	112,478,286	101	110,748,851	98.46%	99.9
BJ2	JM2_ATCACG_L005_R1_001/JM2_ATCACG_L005_R2_001	63,790,670	101	61,969,621	97.15%	98.7
BJ3	JM3_CGATGT_L005_R1_001/JM3_CGATGT_L005_R2_001	55,946,780	101	54,337,396	97.12%	98.7
BJ4	JM4_TTAGGC_L005_R1_001/JM4_TTAGGC_L005_R2_001	92,808,680	101	89,984,188	96.96%	98.6
BJ5	JM5_ACTTGA_L007_R1_001/JM5_ACTTGA_L007_R2_001	98,766,172	101	97,235,566	98.45%	99.9
BJ6	JM6_TGACCA_L005_R1_001/JM6_TGACCA_L005_R2_001	74,893,404	101	72,254,387	96.48%	98.6
BJ7	JM8B_GCCAAT_L005_R1_001/JM8B_GCCAAT_L005_R2_001	82,963,712	101	80,449,239	96.97%	98.7
BJ8	JM8_ACAGTG_L005_R1_001/JM8_ACAGTG_L005_R2_001	53,199,536	101	51,563,810	96.93%	98.6

Settings: Removal of low quality sequence (limit=0.05); removal of ambiguous nucleotides (maximal 2 nt); removal of sequences of minimum length 50 nt.
