# Supplementary material for: Comprehensive transcriptome analysis of early male and female Bactrocera jarvisi embryos
Source: BMC Genet. 2014 Dec 1;15(Suppl 2):S7. doi: 10.1186/1471-2156-15-S2-S7 (PMC4255828; doi:10.1186/1471-2156-15-S2-S7)

Additional File 4. Species distribution of BLAST2GO top matches against a subset of the CLC assembly. A subset of 23,518 contigs were selected by e-value > 1E-3 match to NCBI nr database. Over 77% of the sequences matched *Drosophila* species.

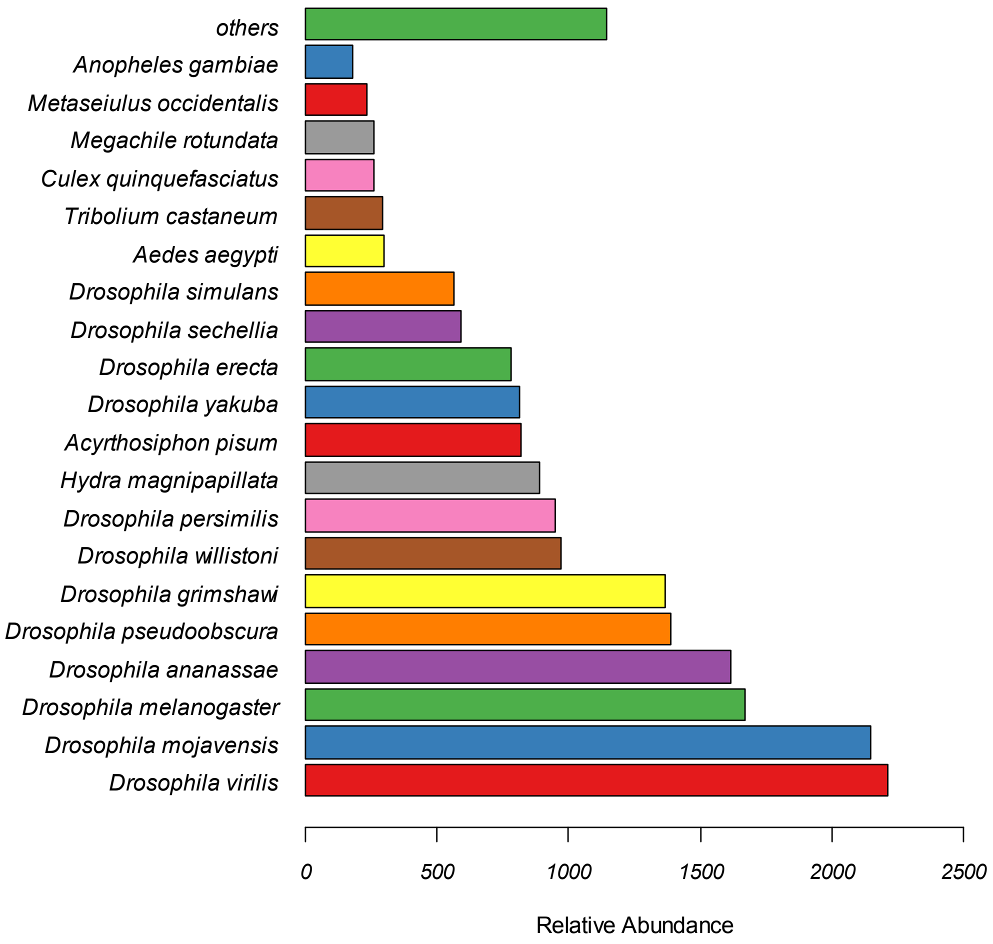

Supplement: Additional File 4 — Species distribution of BLAST2GO top matches against a subset of the CLC assembly. A subset of 23,518 contigs were selected by e-value > 1E-3 match to NCBI nr database. Over 77% of the sequences matched Drosophila species. [file 1471-2156-15-S2-S7-S4.pdf]
