# Supplementary material for: Comprehensive transcriptome analysis of early male and female Bactrocera jarvisi embryos
Source: BMC Genet. 2014 Dec 1;15(Suppl 2):S7. doi: 10.1186/1471-2156-15-S2-S7 (PMC4255828; doi:10.1186/1471-2156-15-S2-S7)
Supplement: Additional File 5 — Sex-determination and cellularisation genes. A) The 28 Drosophila sex determination genes and the top BLAST hits against the nr database. Only 20 of these genes found a match in the CLC assembly. (B) Four cellularisation genes were sought, only nullo was found by the top blast hits. Other transcripts were identified by motif searches using D. melanogaster or C. capitata sequence as the query. [file 1471-2156-15-S2-S7-S5.pdf]

Additional File 5. Sex-determination and cellularisation genes. (A) The 28 *Drosophila* sex determination genes and the top BLAST hits against the nr database. Only 20 of these genes found a match in the CLC assembly. (B) Four cellularisation genes were sought, only nullo was found by the top blast hits. Other transcripts were identified by motif searches using *D. melanogaster* or *C. capitata* sequence as the query.

**A**	**Annotation Symbol**	**contig no.**	**contig size**	**E-value**	**Top blast hit**	**Description**
daughterless	CG5102	16154	4053	0	XP_004521529	PREDICTED: protein daughterless-like [Ceratitis capitata]
deadpan	CG8704	20953	3365	0	XP_004534624	PREDICTED: protein deadpan-like [Ceratitis capitata]
degringolade						no match
dissatisfaction						no match
doublesex	CG11094	22304	1064	2.84E-72	ACN24617	female-specific doublesex protein [Bactrocera dorsalis]
		40032	808	1.35E-58	AAB99947	doublesex [Bactrocera tryoni]
extra-macrochaetae	CG1007	10611	1230	2.02E-67	XP_004525703	PREDICTED: protein extra-macrochaetae-like [Ceratitis capitata]
female lethal d	CG6315	2854	2694	0	XP_004520275	PREDICTED: pre-mRNA-splicing regulator female-lethal(2)D-like isoform X3 [Ceratitis capitata]
fruitless		4880	1862	1.14E-75	XP_004523736	PREDICTED: sex determination protein fruitless-like [Ceratitis capitata]
		29049	3175	4.28E-104	AGL09914	male-specific zinc finger C splice variant [Musca domestica]
groucho	CG8384	3466	4604	0	XP_004518043	PREDICTED: protein groucho-like isoform X1 [Ceratitis capitata]
hermaphrodite						no match
hopscotch	CG1594	753	4443	0	XP_004518856	PREDICTED: tyrosine-protein kinase hopscotch-like [Ceratitis capitata]
intersex						no match
Mes-4	CG4976	5091	5397	0	XP_004530722	PREDICTED: probable histone-lysine N-methyltransferase Mes-4-like isoform X1 [Ceratitis capitata]
Mutant-181						no match
outstretched						no match
ovarian tumor	CG12743	3340	3198	1.41E-72	XP_004527434	PREDICTED: protein ovarian tumor locus-like isoform X3 [Ceratitis capitata]
		5442	201	7.48E-09	XP_004527433	PREDICTED: protein ovarian tumor locus-like isoform X2 [Ceratitis capitata]
		5443	201	1.44E-08	XP_004527433	PREDICTED: protein ovarian tumor locus-like isoform X2 [Ceratitis capitata]
		8947	1757	0	XP_004527434	PREDICTED: protein ovarian tumor locus-like isoform X3 [Ceratitis capitata]
		10161	300	5.23E-24	XP_004527434	PREDICTED: protein ovarian tumor locus-like isoform X3 [Ceratitis capitata]
ovo	CG6824	21175	255	5.10E-26	XP_004527388	PREDICTED: protein ovo-like isoform X1 [Ceratitis capitata]
		22105	317	3.13E-12	XP_004527393	PREDICTED: protein ovo-like isoform X6 [Ceratitis capitata]
		23602	317	3.13E-12	XP_004527393	PREDICTED: protein ovo-like isoform X6 [Ceratitis capitata]
PHD finger protein 7 ortholog						no match

A (cont)	Annotation Symbol	contig no.	contig size	E-value	Top blast hit	Description
runt	CG1849	13344	1521	2.57E-136	XP_004527365	PREDICTED: segmentation protein Runt-like isoform X1 [Ceratitis capitata]
		1493	840	1.33E-26		
		1494	229	9.87E-27		
sans fille	CG4528	7153	1421	4.99E-91	P43332	RecName: Full=U1 small nuclear ribonucleoprotein A; Short=U1 snRNP A; Short=U1-A; Short=U1A; AltName: Full=Sex determination protein snf
scute	CG3827	57147	340	5.11E-57	AAF66944	scute [Ceratitis capitata](389aa)
		50842	514	1.79E-10	AAF66944	scute [Ceratitis capitata]
Sex-lethal	CG43770	2265	1667	1.92E-140	CAG29242	sex-lethal protein [Bactrocera oleae]
Stat92E						no match
sisterless A	CG1641	2508	264	4.66E-09	XP_002055743	sisA [Drosophila virilis] >gi|194150253|gb|EDW65944.1|
					XM_004527132	PREDICTED: Ceratitis capitata protein sisterless A-like (LOC101450422) mRNA
stand still						no match
transformer	CG16724	4523	3261	1.93E-150	XP_004526946	PREDICTED: polycomb group RING finger protein 3-like [Ceratitis capitata]
		13992	392	4.30E-23	AAZ08052	transformer male-specific 1 [Bactrocera oleae]
transformer 2	CG10128	138	3165	6.62E-39	O02008	RecName: Full=Transformer-2 sex-determining protein
virilizer	CG3496	2897	994	1.04E-148	XP_004525965	PREDICTED: LOW QUALITY PROTEIN: protein virilizer-like [Ceratitis capitata]
		4042	392	3.41E-51	XP_004525965	PREDICTED: LOW QUALITY PROTEIN: protein virilizer-like [Ceratitis capitata]
		4266	3049	0	XP_004525965	PREDICTED: LOW QUALITY PROTEIN: protein virilizer-like [Ceratitis capitata]
		4267	1131	1.42E-120	XP_004525965	PREDICTED: LOW QUALITY PROTEIN: protein virilizer-like [Ceratitis capitata]
		5176	234	2.77E-30	XP_004525965	PREDICTED: LOW QUALITY PROTEIN: protein virilizer-like [Ceratitis capitata]
		5177	234	1.71E-30	XP_004525965	PREDICTED: LOW QUALITY PROTEIN: protein virilizer-like [Ceratitis capitata]
		7162	268	9.10E-32	XP_004525965	PREDICTED: LOW QUALITY PROTEIN: protein virilizer-like [Ceratitis capitata]
		7163	268	1.56E-30	XP_004525965	PREDICTED: LOW QUALITY PROTEIN: protein virilizer-like [Ceratitis capitata]
		12608	218	1.72E-12	XP_004525965	PREDICTED: LOW QUALITY PROTEIN: protein virilizer-like [Ceratitis capitata]
		16540	309	2.04E-04	XP_004525965	PREDICTED: LOW QUALITY PROTEIN: protein virilizer-like [Ceratitis capitata]
		16541	310	1.25E-08	XP_004525965	PREDICTED: LOW QUALITY PROTEIN: protein virilizer-like [Ceratitis capitata]

B	Annotation Symbol	contig no.	contig size	E-value	Top blast hit	Description
bottleneck						no match
nullo	CG14462	9415	202	0.18	AAB46422	nullo, partial [Drosophila simulans]
		6453	764	7.82E-06	XP_001978515	GG19631 [Drosophila erecta] >gi|190650164|gb|EDV47442.1|
		14101	1026			no match
		14102	593			no match
		14117	1443			no match
		15641	455			no match
serendipity α	CG17957	9105	5007	0	XP_004536849	PREDICTED: GPI mannosyltransferase 4-like [Ceratitis capitata]
slow as molasses	CG9506	3824	5366	0	XP_004534436	PREDICTED: titin-like [Ceratitis capitata]
