# Supplementary material for: Comprehensive transcriptome analysis of early male and female Bactrocera jarvisi embryos
Source: BMC Genet. 2014 Dec 1;15(Suppl 2):S7. doi: 10.1186/1471-2156-15-S2-S7 (PMC4255828; doi:10.1186/1471-2156-15-S2-S7)
Supplement: Additional File 6 — A list of the 66 contigs up-regulated in older male embryos, after exclusion of contigs also up-regulated in older females. Normalised RPKM values are from comparison I (Table 3) with top BLAST hits to arthropod sequences from the NCBI nr database. [file 1471-2156-15-S2-S7-S6.pdf]

Additional File 6. A list of the 66 arthropod-related contigs up-regulated in older male embryos, after exclusion of contigs also up-regulated in older females. Normalised RPKM values are from experiment I (Table 3) with top BLAST hits to arthropod sequences from the NCBI nr database.

			Normalised RPKM values								Top BLAST Hit	
Contig ID	Contig length	Fold Change	male BJ2	female BJ3	female BJ4	“late” mean	male BJ6	female BJ7	female BJ8	“early” mean	Accession (E-value)	Description
36	665	28.54	88.1	80.1	45.2	71.1	0.7	0.0	6.8	2.5	XP_004525671	PREDICTED: uncharacterized protein LOC101459031 [Ceratitis capitata]
645	589	21.01	25.8	24.7	13.3	21.3	0.3	0.1	2.6	1.0	ABD76335	transposase [Heliothis virescens]
930	564	4.27	62.1	55.1	78.8	65.3	11.7	17.6	16.6	15.3	XP_004521270	PREDICTED: eukaryotic translation initiation factor 3 subunit D-1-like [Ceratitis capitata]
1038	1089	3.54	56.1	52.4	78.0	62.2	13.2	22.6	16.9	17.6	XP_004521155	PREDICTED: uncharacterized protein LOC101455781 [Ceratitis capitata]
1228	2739	4.06	198.5	201.5	131.6	177.2	37.6	45.7	47.7	43.7	XP_004521206	PREDICTED: eukaryotic initiation factor 4A-III-like [Ceratitis capitata]
1817	1774	7.33	75.7	75.5	129.4	93.5	14.1	10.8	13.4	12.8	XP_004537461	PREDICTED: craniofacial development protein 1-like [Ceratitis capitata]
2314	4147	5.98	156.6	162.2	265.5	194.8	23.1	38.9	35.9	32.6	XP_004523499	PREDICTED: DNA primase large subunit-like [Ceratitis capitata]
2876	3809	2.23	115.8	103.0	79.9	99.6	48.6	37.3	47.9	44.6	XP_004521895	PREDICTED: transportin-3-like [Ceratitis capitata]
3007	223	74.82	54.8	66.4	37.3	52.8	0.8	0.0	1.3	0.7	AAB82058	blastoderm-specific protein 25A [Drosophila melanogaster]
3009	1230	13.81	28.5	26.5	14.5	23.2	1.9	0.3	2.8	1.7	XP_001988422	>gi|193904422|gb|EDW03289.1| GH10578 [Drosophila grimshawi]
3156	509	4.16	40.1	35.9	60.7	45.6	9.3	12.6	11.0	11.0	XP_004531652	PREDICTED: calnexin-like [Ceratitis capitata]
3353	982	11.51	88.3	76.9	44.7	70.0	4.6	0.3	13.3	6.1	XP_002066566	>gi|194162651|gb|EDW77552.1| GK24494 [Drosophila willistoni]
3466	4604	6.37	51.4	59.5	94.4	68.4	7.4	13.3	12.7	11.1	XP_004518043	PREDICTED: protein groucho-like isoform X1 [Ceratitis capitata]
3515	2772	4.26	88.8	92.8	135.3	105.6	23.1	25.1	26.2	24.8	XP_004529388	PREDICTED: keratin, type I cytoskeletal 9-like [Ceratitis capitata]
3878	3339	46.38	73.1	85.2	46.1	68.1	1.2	1.1	2.2	1.5	XP_004521898	PREDICTED: TNF receptor-associated factor 4-like isoform X1 [Ceratitis capitata]
4660	4268	14.5	133.7	134.4	213.3	160.5	5.9	14.1	13.2	11.1	XP_004529208	PREDICTED: histone-arginine methyltransferase CARMER-like [Ceratitis capitata]
4679	1184	13.79	256.9	192.6	126.5	192.0	7.0	0.6	34.2	13.9	XP_004522978	PREDICTED: poly(A) polymerase alpha-like [Ceratitis capitata]
4783	326	15.69	261.6	263.9	165.5	230.4	3.6	15.1	25.3	14.7	XP_004517515	PREDICTED: F-box/LRR-repeat protein 14-like isoform X2 [Ceratitis capitata]
4784	2318	10.95	409.6	336.4	257.4	334.5	12.6	34.2	44.9	30.6	XP_004517511	PREDICTED: F-box/LRR-repeat protein 13-like [Ceratitis capitata]
4801	3454	3.65	123.3	124.4	87.4	111.7	25.2	27.6	39.0	30.6	XP_004534997	PREDICTED: polymerase delta-interacting protein 3-like [Ceratitis capitata]
4934	2359	14.06	163.4	160.5	96.1	140.0	7.4	7.2	15.3	10.0	XP_004535595	PREDICTED: parafibromin-like [Ceratitis capitata]
4984	2762	20.4	320.0	302.8	176.1	266.3	8.0	8.6	22.6	13.1	XP_001988422	>gi|193904422|gb|EDW03289.1| GH10578 [Drosophila grimshawi]
5000	665	9.25	66.8	50.0	33.7	50.2	1.2	0.2	14.9	5.4	XP_004524168	PREDICTED: protein toll-like [Ceratitis capitata]

			Normalised RPKM values								Top BLAST Hit	
**Contig ID**	**Contig length**	**Fold Change**	**male BJ2**	**female BJ3**	**BJ4**	**“late” mean**	**male BJ6**	**female BJ7**	**BJ8**	**“early” mean**	**Accession (E-value)**	**Description**
6083	406	44.33	139.2	150.8	85.2	125.1	1.9	1.0	5.5	2.8	AAB82058	blastoderm-specific protein 25A [Drosophila melanogaster]
6152	629	8.26	183.9	153.9	93.4	143.7	7.8	15.7	28.8	17.4	XP_004518482	PREDICTED: serine/arginine repetitive matrix protein 2-like [Ceratitis capitata]
6356	793	16.87	48.5	54.6	32.4	45.2	2.4	0.1	5.5	2.7	XP_001355983	>gi|54644301|gb|EAL33042.1| GA22097 [Drosophila pseudoobscura pseudoobscura]
6406	256	14.23	35.9	26.3	16.6	26.3	2.4	0.4	2.7	1.9	XP_004519076	PREDICTED: uncharacterized protein LOC101456548 [Ceratitis capitata]
6564	2000	17.45	162.9	137.6	81.6	127.4	1.6	5.4	14.9	7.3	XP_004537817	PREDICTED: mediator of RNA polymerase II transcription subunit 15-like [Ceratitis capitata]
7120	2686	51.74	22.1	19.6	10.0	17.2	0.2	0.1	0.7	0.3	XP_004530768	PREDICTED: uncharacterized protein LOC101463003 [Ceratitis capitata]
7485	1504	8.07	79.5	70.8	42.6	64.3	3.4	8.0	12.6	8.0	XP_004522803	PREDICTED: ATP-dependent RNA helicase DHX8-like isoform X2 [Ceratitis capitata]
7654	216	3.9	65.5	59.7	86.3	70.5	14.9	13.5	25.9	18.1	XP_004517756	PREDICTED: eukaryotic translation initiation factor 3 subunit B-like [Ceratitis capitata]
7816	201	3.88	33.6	24.7	31.8	30.0	4.9	6.5	11.8	7.7	XP_004521644	PREDICTED: protein BUD31 homolog [Ceratitis capitata]
8666	3357	8.49	82.9	82.8	137.3	101.0	6.8	15.6	13.3	11.9	XP_004534405	PREDICTED: protein ref(2)P-like [Ceratitis capitata]
8873	431	3.32	29.7	28.5	23.6	27.3	4.5	8.5	11.7	8.2	XP_004520278	PREDICTED: pre-mRNA-splicing factor SYF1-like [Ceratitis capitata]
8897	2006	17.81	38.9	36.1	18.0	31.0	0.3	0.1	4.8	1.7	ACO12003	Transposable element Tc3 transposase [Lepeophtheirus salmonis]
8928	881	2.9	107.5	91.4	121.2	106.7	25.5	41.6	43.2	36.8	XP_004521190	PREDICTED: mitotic checkpoint protein BUB3-like [Ceratitis capitata]
10611	1230	126.7	121.3	123.6	211.0	152.0	0.4	0.6	2.5	1.2	XP_004525703	PREDICTED: protein extra-macrochaetae-like [Ceratitis capitata]
10944	2255	8.96	47.5	43.7	24.7	38.6	1.6	5.2	6.1	4.3	XP_004522803	PREDICTED: ATP-dependent RNA helicase DHX8-like isoform X2 [Ceratitis capitata]
11509	4304	15.09	25.9	30.9	14.8	23.8	0.6	2.1	2.0	1.6	XP_004519049	PREDICTED: la-related protein CG11505-like isoform X5 [Ceratitis capitata]
11846	281	11.63	31.6	23.9	15.3	23.6	0.8	0.0	5.3	2.0	EFN64458	PiggyBac transposable element-derived protein 4 [Camponotus floridanus]
11944	1366	3.56	36.8	29.8	51.3	39.3	6.6	13.6	12.9	11.1	XP_004533749	PREDICTED: ATPase ASNA1 homolog [Ceratitis capitata]
12135	1736	3.95	63.7	61.8	89.9	71.8	13.9	20.8	19.8	18.2	XP_004521155	PREDICTED: uncharacterized protein LOC101455781 [Ceratitis capitata]
12338	378	91.93	35.1	43.7	75.1	51.3	0.9	0.3	0.5	0.6	XP_004526058	PREDICTED: UDP-glucuronosyltransferase 2B15-like [Ceratitis capitata]
12582	669	33.81	39.1	37.0	21.9	32.7	0.2	0.2	2.6	1.0	XP_004213206	PREDICTED: piggyBac transposable element-derived protein 4-like [Hydra magnipapillata]
13120	350	20.91	52.8	38.2	23.5	38.2	1.7	0.2	3.6	1.8	XP_004522172	PREDICTED: zygotic gap protein knirps-like isoform X1 [Ceratitis capitata]
13344	1521	29.64	54.3	55.0	27.7	45.7	0.3	0.0	4.3	1.5	XP_004527365	PREDICTED: segmentation protein Runt-like isoform X1 [Ceratitis capitata]

			Normalised RPKM values								Top BLAST Hit	
Contig ID	Contig length	Fold Change	male BJ2	female BJ3	female BJ4	"late" mean	male BJ6	female BJ7	female BJ8	"early" mean	Accession (E-value)	Description
13492	551	5.8	64.6	56.9	38.3	53.3	5.4	11.7	10.5	9.2	XP_004530499	PREDICTED: probable serine/threonine-protein kinase yakA-like [Ceratitis capitata]
13588	1070	38.38	40.3	40.0	20.7	33.7	0.3	0.5	1.8	0.9	NP_001266341	>gi|214028056|gb|ABW97511.1| alpha-esterase 7 [Ceratitis capitata]
13723	701	32.3	46.3	41.8	21.9	36.7	0.4	0.8	2.2	1.1	NP_001266341	>gi|214028056|gb|ABW97511.1| alpha-esterase 7 [Ceratitis capitata]
14900	337	2.72	57.2	50.2	34.5	47.3	13.1	19.3	19.8	17.4	XP_004522151	PREDICTED: DNA-directed RNA polymerase III subunit RPC4-like isoform X2 [Ceratitis capitata]
15106	2560	2.59	46.9	41.5	32.6	40.3	10.9	17.5	18.4	15.6	XP_004531488	PREDICTED: protein sly1 homolog [Ceratitis capitata]
15168	2974	135.45	25.9	18.0	40.4	28.1	0.2	0.1	0.3	0.2	AAN87269	ORF [Drosophila melanogaster]
15265	1176	49.3	28.3	27.8	16.6	24.3	0.2	0.1	1.2	0.5	NP_476730	>gi|7295755|gb|AAF51058.1| sloppy paired 1 [Drosophila melanogaster]
15562	1261	5.89	37.0	36.2	54.4	42.6	2.8	10.0	8.8	7.2	XP_004534447	PREDICTED: uncharacterized protein LOC101461354 isoform X1 [Ceratitis capitata]
15731	492	12.97	23.8	19.4	13.1	18.8	0.6	0.0	3.7	1.5	XP_002060047	GJ15515 [Drosophila virilis] >gi|194141845|gb|EDW58258.1| GJ15515 [Drosophila virilis]
16200	360	4.2	31.5	23.3	22.1	25.6	1.4	8.4	8.6	6.1	XP_004520506	PREDICTED: cyclin-H-like [Ceratitis capitata]
16518	570	4.11	47.9	42.0	69.0	52.9	12.3	10.1	16.2	12.9	XP_004530540	PREDICTED: host cell factor-like [Ceratitis capitata]
16546	2732	6.16	36.9	31.8	21.1	29.9	2.8	5.0	6.8	4.9	XP_004537763	PREDICTED: inhibitor of growth protein 3-like [Ceratitis capitata]
16608	2306	6.82	36.6	31.5	53.4	40.5	2.3	8.8	6.7	5.9	XP_004525046	PREDICTED: protein DDI1 homolog 2-like isoform X1 [Ceratitis capitata]
17055	222	14.79	142.4	107.2	71.8	107.1	4.4	0.0	17.3	7.2	XP_004523465	PREDICTED: protein spaetzle-like isoform X2 [Ceratitis capitata]
18109	924	3.62	26.3	21.0	20.2	22.5	2.5	7.8	8.3	6.2	XP_004535038	PREDICTED: carbonic anhydrase 2-like isoform X1 [Ceratitis capitata]
19290	698	5.6	51.7	43.4	68.8	54.6	1.6	14.2	13.5	9.8	XP_004518396	PREDICTED: DNA polymerase V-like [Ceratitis capitata]
20291	270	9.57	36.1	37.7	19.7	31.2	2.1	3.4	4.2	3.3	XP_004522802	PREDICTED: ATP-dependent RNA helicase DHX8-like isoform X1 [Ceratitis capitata]
20341	1943	7.31	23.3	15.8	27.4	22.2	1.1	4.1	3.9	3.0	XP_004526995	PREDICTED: serine-rich adhesin for platelets-like isoform X1 [Ceratitis capitata]
20639	1028	17	38.7	36.7	63.1	46.2	2.4	2.3	3.5	2.7	XP_004536159	PREDICTED: kelch-like protein 5-like [Ceratitis capitata]
23008	1377	4.88	35.4	28.2	47.8	37.1	2.0	10.8	10.1	7.6	XP_004518396	PREDICTED: DNA polymerase V-like [Ceratitis capitata]
