# Supplementary material for: Comprehensive transcriptome analysis of early male and female Bactrocera jarvisi embryos
Source: BMC Genet. 2014 Dec 1;15(Suppl 2):S7. doi: 10.1186/1471-2156-15-S2-S7 (PMC4255828; doi:10.1186/1471-2156-15-S2-S7)
Supplement: Additional File 7 — A list of the Bactrocera jarvisi contigs up-regulated in older embryos with BLAST matches to C. capitata and Drosophila genes (62 contigs). These were cross-referenced to FlyBase. The molecular and biological function of these genes and the expression profile in D. melanogaster are listed. Many genes have transcription or translation regulatory functions, but are transcribed in both males and females (see Additional File 6). [file 1471-2156-15-S2-S7-S7.pdf]

Additional file 7. A list of the *Bactrocera jarvisi* contigs up-regulated in older embryos with BLAST matches to *C. capitata* and *Drosophila* genes (62 contigs). These were cross-referenced to FlyBase. The molecular and biological function of these genes and the expression profile in *D. melanogaster* are listed. Many genes have transcription or translation regulatory functions, but are transcribed in both males and females (see Additional File 6).

	Closest *Drosophila* match			
**contig ID**	**Annotation ID**	**Gene name**	**Molecular or biological function**	**Expression profile**
36	CG7163	mkg-p, monkey-king protein	nucleotidyltransferase activity	maternal
930	CG10161	eiF-3p66, Eukaryotic initiation factor 3 p66 subunit	translation initiation factor activity	
1038	CG9684		mRNA splicing, via spliceosome	0-6h, adult female
1228	CG7483	eIF4AIII	ATP binding; protein binding; translation initiation factor activity; ATP-dependent RNA helicase activity	0-18h
1817	CG40218	Yeti	kinesin binding	0-6h, 12-18h
2314	CG7878		ATP binding; RNA binding; ATP-dependent RNA helicase activity; helicase activity	0-6h, maternal, adult females.
2876	CG2848	Trn-SR, Transportin-Serine/Arginine rich	Ran GTPase binding; protein binding	0-12h, maternal, adult female reproductive system.
3007	CG12205	Bsg25A, Blastoderm-specific gene 25A	sequence-specific DNA binding; chromatin insulator sequence binding	Detected during nuclear cycle 10-13
3009	CG12205	Bsg25A, Blastoderm-specific gene 25A	sequence-specific DNA binding; chromatin insulator sequence binding	Detected during nuclear cycle 10-13
3156	CG11958	Cnx99A, Calnexin 99A	calcium ion binding; unfolded protein binding	0-18h
3353	CG3227	insv, insensitive	transcription corepressor activity	0-6h, maternal
3466	CG8384	gro, groucho	RNA polymerase II transcription corepressor activity; transcription corepressor activity; transcription factor binding; protein binding; repressing transcription factor binding; HMG box domain binding	0-6h, maternal
3515	CG6605	Bicaudal D	protein binding	0-6h and adult females, maternal, rapidly degraded stage 4-6
3878	CG3048	TNF receptor-associated factor 4	zinc-finger protein binding	0-12h, stage4-6
4660	CG5358	Arginine methyltransferase 4 (Art4)	histonearginine N-methyltranferase activity	0-6h and adult female, maternal
4679	CG9854	hrg, hiragi	polynucleotide adenylyltransferase activity; RNA binding	0-6h, maternal, rapidly degraded at stage 4-6
4783	CG1839	Fbxl4, Fbox and leucine-rich-repeat gene4		0-12h
4784	CG1839	Fbxl4, Fbox and leucine-rich-repeat gene4		0-12h
4801	CG18259		nucleotide binding; nucleic acid binding	0-12h
4934	CG3227	insv, insensitive	transcription corepressor activity	0-6h, maternal
4984	CG3227	insv, insensitive	transcription corepressor activity	0-6h, maternal
5000	CG5490	Toll	protein binding	embryo stage 14-16 and pupae

	Closest *Drosophila* match			
contig ID	Annotation ID	Gene name	Molecular or biological function	Expression profile
6083	CG12205	Bsg25A, Blastoderm-specific gene 25A	sequence-specific DNA binding; chromatin insulator sequence binding	Detected during nuclear cycle 10-13
6152	CG9775			0-12h, maternal
6356	CG9883	Elba2 Early boundary activity 2	chromatin insulator sequence binding; sequence specific binding	0-6h, maternal, present adult ovary,
6406	CG13713		regulation of localization	0-6h
6564	CG4184	MED15, Mediator complex subunit 15	regulation of transcription from RNA polymerase II promoter; transcription initiation from RNA polymerase II promoter	0-6h, maternal
7120	CG14064	beat-VI	heterophilic cell-cell adhesion	12-24h
7485	CG8241	pea, peanuts	ATP binding; RNA binding; regulation of alternative mRNA splicing, via spliceosome	0-6h, adult ovary
7654	CG4878	elF3-S9	mRNA binding; translation initiation factor activity; nucleotide binding; translation initiation factor binding	maternal
7816	CG1639	l(1)10Bb, lethal (1) 10Bb		0-6h, maternal
8666	CG10360	ref(2)P, refractory to sigma P	predicted zincion binding	0-6h, maternal, male and female adults,
8873	CG6197		regulation of alternative mRNA splicing, via spliceosome	0-6h, adult ovary
8928	CG7581	Bub3	mitotic cell cycle checkpoint, mitotic spindle organisation and mitotic spindle assembly checkpoint	0-12h, maternal
10611	CG1007	emc, extra-macrochaetae	protein binding transcription factor activity, protein heterodimerisation activity, predict transcription corepressor activity	0-18h, maternal
10944	CG8241	pea, peanuts	ATP binding; RNA binding; regulation of alternative mRNA splicing, via spliceosome	0-6h, adult ovary
11509	CG11505		1 HTH La-type RNA-binding domain, predicted nucleic acid binding, nucleotide binding	0-6h, adult ovary and testis
11944	CG1598		ATP binding; arsenite-transmembrane transporting ATPase activity	0-6h, maternal
12135	CG9684		mRNA splicing, via spliceosome	0-6h, adult female
12338	CG15661		glucuronosyltransferase activity	6-24h
13120	CG4717	kni, knirps	repressing transcription factor binding, sequence-specific DNA binding transcription factor activity	0-6h, maternal
13344	CG1849	runt	sequence-specific DNA binding transcription factor activity; ATP binding	0-12h, zygotic
13492	CG12701	vfl, vielfaltig (also zld, zelda)	sequence-specific DNA binding; metal ion binding; transcription regulatory region sequence-specific DNA binding	0-12h, maternal, adult ovary
13588	CG1112	a-Est7, a-Esterase-7	carboxylesterase activity	embryo stage 11, adult male

contig ID	Annotation ID	Gene name	Molecular or biological function	Expression profile
	Closest *Drosophila* match			
13723	CG1112	a-Est7, a-Esterase-7	carboxylesterase activity	embryo stage 11, adult male
14900	CG5147		DNA binding; DNA-directed RNA polymerase activity	0-18h
15106	CG3539	Slh, SLY-1 homologous	predicted SNARE binding	maternal
15168	na	roo/ORF, ORF	predicted RNA-binding, RNA-directed DNA polymerase activity, zinc ion binding	
15265	CG16738	slp1, sloppy paired 1	sequence-specific DNA binding transcription factor activity	0-12h, first expressed stage 4
15562	CG40191		protein kinase binding	6-12h
15731	CG13713			0-6h, zygotic
16200	CG7405	cycH, Cyclin H	RNA polymerase II carboxy-terminal domain kinase activity; protein kinase binding	0-12h, adult female
16518	CG1710	Hcf, Host cell factor	sequence-specific DNA binding transcription factor activity; histone acetyltransferase activity; chromatin binding; transcription coactivator activity	embryogenesis, maternal, adult female
16546	CG42638		Polycystin cation channel	embryogenesis, adults
16608	CG4420	rngo. Rings lost	proteasome binding; ubiquitin binding; aspartic-type endopeptidase activity	0-12h, maternal, adult female
17055	CG6134	spz, spatzle	morphogen activity; protein homodimerization activity; cytokine activity; growth factor activity; Toll binding	0-6h, maternal, female adult
18109	CG11284		carbonate dehydratase activity	0-6h, adult females
19290	CG6189	l(1)1Bi, lethal (1) 1Bi	DNA binding; DNA-directed DNA polymerase activity	embryogenesis, early larval, adult female
20291	CG8241	pea, peanuts	ATP binding; RNA binding; regulation of alternative mRNA splicing, via spliceosome	0-6h, adult ovary
20341	CG33232		actin binding	0-6h, adult female reproductive system
20639	CG7210	kel, kelch	actin binding	0-6, 18-24h, maternal
23008	CG6189	l(1)1Bi, lethal (1) 1Bi	DNA binding; DNA-directed DNA polymerase activity	embryogenesis, early larval, adult female
